# Absence of pain in subjects with advanced radiographic knee osteoarthritis

**DOI:** 10.1186/s12891-020-03647-x

**Published:** 2020-09-29

**Authors:** Kyeong Min Son, Jeong Im Hong, Dong-Hyun Kim, Dae-Gyu Jang, Michel D. Crema, Hyun Ah Kim

**Affiliations:** 1 Division of Rheumatology, Department of Internal Medicine, Hallym University Dongtan Sacred Heart Hospital, 896, Pyongchon, Hwaseong-si, Anyang, Kyunggi 431-070 South Korea; 2 Division of Rheumatology, Department of Internal Medicine, Hallym University Sacred Heart Hospital, Anyang, Kyunggi South Korea; 3 Deparment of Social and Preventive Medicine, Hallym University College of Medicine, Chuncheon, Kangwon South Korea; 4 Department of Statistics, Seoul National University, Gwanak, Seoul, South Korea; 5 Department of Radiology, Boston University School of Medicine, Boston, Massachusetts USA

**Keywords:** Osteoarthritis, Pain, Knee

## Abstract

**Background:**

To investigate the frequency of pain among subjects with advanced radiographic knee osteoarthritis (OA) defined as Kellgren–Lawrence (KL) grade 4 and clinical features associated with pain.

**Methods:**

Subjects from the Hallym Aging Study (HAS), the Korean National Health and Nutrition Examination Survey (KNHANES), and the Osteoarthritis Initiative (OAI) were included. Participants were asked knee-specific questions regarding the presence of knee pain. Clinical characteristics associated with the presence of pain were evaluated with multivariable logistic regression analysis.

**Results:**

The study population consisted of 504, 10,152 and 4796 subjects from HAS, KNHANES, and OAI, respectively. KL grade 4 OA was identified in 9.3, 7.6, and 11.5% of subjects, while pain was absent in 23.5, 31.2, and 5.9% of subjects in KL grade 4 knee OA, respectively. After multivariable analysis, female gender showed a significant association with pain in the KNHANES group, while in the OAI group, younger age did. Advanced knee OA patients without pain did not differ from non-OA subjects in most items of SF-12 in both Korean and OAI subjects. Total WOMAC score was not significantly different between non-OA and advanced knee OA subjects without pain in the OAI.

**Conclusions:**

Our study showed that a considerable number of subjects with KL grade 4 OA did not report pain. In patients whose pain arises from causes other than structural damage of the joint, therapeutic decision based on knee X-ray would lead to suboptimal result. In addition, treatment options focusing solely on cartilage engineering, should be viewed with caution.

## Background

Osteoarthritis (OA) is the most common form of arthritis affecting the elderly, and was ranked as the 11th leading cause of years lived with disability (YLD) globally, with higher rank (6th) especially among Asian countries [1]. In addition, YLD due to knee OA increased by 64% from 1990 to 2010, reflecting aging of the population [1]. Knee pain due to OA is a key symptom influencing the decision to seek medical attention, and previous reports have suggested that knee pain is a better predictor of disability than radiographic changes [2, 3]. Radiographic OA changes are poorly correlated with pain and physical function, and the risk factors for radiographic knee OA may not be the same as those for knee pain [4–6]. Although radiography has long been used for evaluation of knee OA and knee pain, caution is required in its use to guide therapeutic decisions, such as whether to perform knee replacement surgery.

The mechanistic causes of pain in knee OA is not well understood, although multiple factors, including biological and psychological causes, may all play important roles. Hyaline cartilage, the main focus of interest in both clinical and laboratory research on OA, is an aneural structure [7]. Thus, while clinicians tend to relate structural damage to the cartilage in people with knee pain, it is unable to provide sensory nociceptive input and is, therefore, an unlikely source of pain. Radiographic knee OA accompanied with pain occurs in approximately 37% of persons aged 60 years or older, whereas knee pain is reported at a rate of 15–81% among subjects with radiographic knee OA [6, 8–11]. The discordance between knee pain and knee radiographic findings was suggested to be more prominent in cases of mild grade OA. However, discordance was also reported in Kellgren–Lawrence (KL) grade 3 or 4, with 25.8% of subjects reporting no pain in the knee [6, 12, 13]. KL grade 4 OA represents the most severe subgroup of radiographic OA, showing marked joint space narrowing (often bone-to-bone appearance reflecting almost total loss of cartilage), large osteophytes, severe subchondral sclerosis, and definite bony deformity. Patients presenting with knee pain and KL grade 4 radiographs are more likely to be offered joint replacement surgery, because a previous cohort study showed that a higher degree of radiographic OA was related to shorter time until surgery, and because it was consistently reported that patients with higher grade OA have a better prognosis after total hip replacement (THR) or total knee replacement (TKR) than patients with lower grade OA [14, 15]. In patients whose pain arises from causes other than structural damage of the joint, however, decision of surgery solely based on knee X-ray would lead to suboptimal result.

This study was performed to elucidate the frequency of pain among subjects with advanced radiographic knee OA defined as KL grade 4 OA. Clinical characteristics associated with pain among advanced radiographic knee OA subjects were examined. In addition, knee function and quality of life among non-OA, advanced radiographic knee OA without pain and with pain were compared. Two databases obtained from Korean community residents and the Osteoarthritis Initiative (OAI) public-use data sets were used and analyzed.

## Methods

### Subjects

Subjects were from two Korean registries, i.e., the Hallym Aging Study (HAS) and the fifth Korean National Health and Nutrition Examination Survey (KNHANES-V, 2010–2012), and the OAI. HAS is a prospective cohort study of health among elderly community residents of Chuncheon, a city about 120 km east of Seoul. The methods have been described elsewhere in detail [3]. Briefly, eligibility criteria included age ≥ 50 years and residence within the borders of the survey area for at least 6 months before the survey. This study began in 2004, with follow-up examinations planned every 3 years until 2010, and the study population included subjects recruited in 2007. KNHANES was a cross-sectional and nationally representative survey conducted by the Division of Chronic Disease Surveillance of the Korean Center For Disease Control and Prevention [16]. This survey used a stratified, multistage, clustered sampling design based on the 2005 National Census data to randomly select a population based across 500 national districts to represent the civilian, non-institutionalized, South Korean population, and sample design and size were estimated such that the annual survey results were representative of the whole population in Korea [17]. In the present study, we selected all subjects ≥50 years (*n* = 10,152) who had an OA examination that included radiographic examination of the knee and a survey about knee pain [18]. The OAI is a nationwide research study sponsored by the National Institutes of Health. The OAI study included men and women aged 45–79 years with, or at risk of, symptomatic tibiofemoral knee OA, to investigate the natural history of knee OA across the spectrum of disease and to study the relationships of imaging, and biochemical and genetic markers with the clinical course of OA. We used the baseline questionnaire for the cohort of 4796 participants from November 2008, which is publicly accessible at https://data-archive.nimh.nih.gov/oai.

Demographic information included in the three surveys were sex, age, body mass index (BMI), education level, marital status, smoking history, alcohol consumption, household income, and comorbidities. Comorbidity health information was obtained using a self-reported questionnaire survey (see Additional file 1) and included cerebrovascular accident (CVA), myocardial infarction, diabetes mellitus, hyperlipidemia (not available in the OAI groups), and osteoporosis.

### Definition of knee pain

In the HAS study, knee pain was assessed by asking, “Have you had pain, aching, or stiffness lasting at least a month in your knee?” In the KNHANES study, knee pain was assessed by asking, “Have you had an episode of knee pain lasting more than 30 days within the last 3 months?” In OAI, knee pain was assessed by asking, “During the past 12 months, have you had pain, aching, or stiffness in or around your right (left) knee on most days for at least one month?” Subjects without knee pain were defined as those who responded “No” to the screening question in the cohort.

### WOMAC score

WOMAC score is the most commonly used disease-specific measure of outcome in OA. The WOMAC score was based on the objective of defining the dimensionality of pain and disability in osteoarthritis of the hip and/or knee [19–21]. WOMAC score for knee pain was collected in HAS and OAI. In HAS, WOMAC score was based on a 0–100-mm visual analog scale, and the total score ranged from 0 to 2400. In the OAI study, WOMAC score was based on categorical score using the Likert scale and total score ranged from 0 to 96.

#### SF-12

To assess general health status, the self-administered Medical Outcomes Study Short Form 12 (SF-12) was used, which is a reduced version of the SF-36 in that it has the same number of subscales, but with fewer items per subscale. Scores are derived from 8 domains and higher scores represent better health [22].

### Radiographic assessment

In the HAS study, radiographic evaluations consisted of bilateral weight-bearing anteroposterior (AP), semi-flexed knee radiographs, using a Plexiglas frame (SYNARC, San Francisco, CA) to standardize knee positions, according to the manufacturer’s recommendations. Radiographic grade of knee OA was assessed using the KL grading and were read twice by one reader (an academically based rheumatologist). The reproducibility of intrareader assessment was high (for OA vs. no OA, weighted Cohen’s κ = 0.89). In the KNHANES study, bilateral weight-bearing AP knee radiographs were taken using a SD3000 Synchro Stand (Accele Ray Shinyoung Co., Seoul, Korea). Radiographic changes related to OA were assessed using the KL grading system. The radiographic digital images were graded by two radiologists, with concordant grades accepted. When there was a difference of 1 grade between the two radiologists, the higher grade was accepted. If the discrepancy was > 1 grade, a third radiologist was consulted, and the grade concordant with the third grader was accepted [23]. Inter-rater agreement within 1 grade of difference between the two radiologists was 92.8% and weighted Cohen’s κ coefficient was 0.65.In OAI, bilateral posterior–anterior (PA) fixed flexion view was taken. Readers at each clinical center were trained using a classification based on the OARSI atlas grades, and had to achieve acceptable agreement with the central reading. In the present study, the definition of advanced radiographic knee OA was the presence of KL grade 4 in any tibiofemoral joint. The definition of non-OA was the KL grade 0 regardless the knee pain.

### Statistical analysis

To compare three groups (HAS, KNHANES, and OAI), continuous variables were examined using ANOVA or Kruskal Wallis test and categorical variables using the chi-square test. To compare advanced radiographic knee OA subjects with pain to those without pain, continuous variables were tested using two-sample *t* test or Mann–Whitney *U* test and categorical variables were examined using the chi-square test. In multivariable analysis for risk factors of knee pain, we used ordinal regression analysis with the backward selection method with all clinical factors (sex, age, BMI, education level, marital status, smoking history, alcohol consumption, household income, and comorbidities). We used the backward selection method since it is one of the most widely used methods in clinical studies. We also used forward and stepwise selection methods, and selected variables in the model were the same for each dataset.

Crude odds ratio (OR) for risk factors of knee pain in advanced radiographic knee OA were calculated using the 95% confidence intervals (CI). Data were analyzed using SAS (SAS Institute Inc., Cary, NC). All data are presented as the means and standard deviations (SD) if they had a normal distribution and median and interquartile range (IQR) if not, or as percentages. In all analyses, *P* < 0.05 (2-tailed) was taken to indicate statistical significance.

### Ethics statement

The HAS study and KNHANES study were approved by the institutional review board of the Hallym University School of Medicine (IRB approval number: HIRB-2007-001) and by the Korea Centers for Disease Control and Prevention institutional review board in Korea (2010-02CON-21-C, 2011-02CON-06-C and 2012-01EXP-01-2C), respectively. Both study protocols conformed to the ethical guidelines of the 1975 Declaration of Helsinki and written informed consent was obtained from each participant.

## Results

The characteristics of the subjects included in the HAS, KNHANES, and OAI are shown in Table 1. The HAS participants were the oldest, followed by those in KNHANES and OAI with mean ages of 70.1, 64.3, and 61.1 years, respectively. The Korean surveys included more male subjects compared to OAI (54.3 and 56.9% female in HAS and KNHANES, respectively, compared 58.5% in OAI). In addition, Korean subjects had lower BMI compared to OAI. The percentages of advanced radiographic knee OA (KL grade 4) were 9.3, 7.6, and 11.5% in HAS, KNHANES, and OAI, respectively. Among Korean subjects with advanced OA, 23. 5 and 31.2% in HAS and KNHANES, respectively, did not report pain, while the rate was 5.9% among OAI subjects.

**Table 1:** Baseline characteristics of the three study groups

Variables	HAS(*N* = 504)	KNHANES(*N* = 10,152)	OAI(*N* = 4796)	*P*-value
Age (years), means ± SD	70.1 ± 7.9	64.3 ± 9.5	61.1 ± 9.1	< 0.0001
Female	54.3	56.9	58.5	0.0774
Diabetes	10.1	14.6	7.7	< 0.0001
CVA	6.3	3.4	2.9	0.0003
MI	4.5	5.1	1.9	< 0.0001
Hyperlipidemia	5.7	16.3	NA	< 0.0001
Osteoporosis	19.2	12.3	12.1	< 0.0001
BMI (kg/m^2^), means ± SD	24.6 ± 3.2	23.9 ± 3.1	28.6 ± 4.8	< 0.0001
Smoker	40.4	40.5	47.1	< 0.0001
Alcohol	41.4	77.5	80.3	< 0.0001
Low education	77.9	65.2	70.9	< 0.0001
Low income	45.9	75.2	75.7	< 0.0001
Marriage	68.8	77.7	66.8	< 0.0001
KL grade 4 in any tibio-femoral joint	9.3	7.6	11.5	< 0.0001

All data are presented as percentages unless otherwise specified. Low education level was defined as < 9 years of education in HAS and KNHANES, and as less than high school graduation in OAI. Low income was defined as the lowest quartile of yearly household income in HAS and KNHANES and as yearly household income lower than $100,000 USD in OAI. Continuous variables showing normal distribution (BMI) were tested using ANOVA. Age was tested using Kruskal Wallis test and categorical variables using chi-square test

*HAS* Hallym Aging Study, *KNHANES* Korean National Health and Nutrition Examination Survey, *OAI* Osteoarthritis Initiative, *SD* Standard deviation, *CVA* Cerebrovascular accident, *MI* Myocardial infarction, *KL grade* Kellgren–Lawrence grade

We compared the clinical characteristics of advanced radiographic knee OA subjects with pain to those without pain. In both Korean groups, the proportion of female subjects was higher in those with pain compared to those without pain (86.1% vs. 54.5%, *P* = 0.03 and 87.9% vs. 77.1%, *P* = 0.0003, in HAS and KNHANES, respectively) (Table 2). In addition, the rate of smokers was lower and the rate of lower education level was higher among subjects with knee pain in the KNHANES group. While there was no difference in age between asymptomatic and symptomatic subjects in Korean surveys, symptomatic subjects were significantly younger compared to asymptomatic subjects in OAI. In addition, symptomatic subjects had a higher rate of osteoporosis in the Korean groups, while the reverse was true for OAI.

**Table 2:** Comparison of clinical characteristics of advanced radiographic knee OA subjects in three study groups

	HAS			KNHANES			OAI		
Variables	Without pain (*N* = 11)	With pain (*N* = 36)	*P* value	Without pain (*N* = 214)	With pain (*N* = 471)	*P* value	Without pain (*N* = 33)	With pain (*N* = 521)	*P* value
Age (years), means ± SD	76.8 ± 8.9	72.3 ± 6.8	0.09	72.2 ± 8.0	72.2 ± 7.8	0.71	**70.3 ± 7.6**	**64.2 ± 8.7**	**<.0001**
Female	**54.5**	**86.1**	**0.03**	**77.1**	**87.9**	**0.0003**	48.5	52.4	0.66
Diabetes	9.0	11.1	0.85	15.8	19.7	0.23	12.5	9.1	0.52
CVA	0	5.5	0.42	1.8	4.2	0.12	3.1	3.3	0.95
MI	0	5.5	0.42	4.6	5.3	0.73	3.2	1.9	0.63
Hyperlipidemia	0	0	NA	11.2	16.5	0.07	NA	NA	NA
Osteoporosis	9.0	27.7	0.2	**14.2**	**27.6**	**0.0041**	**24.2**	**11.4**	**0.03**
BMI (kg/m^2^), means ± SD	26.3 ± 3.8	26.3 ± 4.6	0.98	25.0 ± 3.8	25.2 ± 3.8	0.58	29.2 ± 4.1	29.8 ± 4.5	0.5
Smoker	18.1	25.0	0.64	**25.8**	**16.2**	**0.0033**	37.5	49.7	0.18
Alcohol	45.4	38.8	0.7	63.3	60.4	0.47	78.1	82.2	0.56
Low education	100	94.4	0.42	**85.9**	**94.9**	**<.0001**	96.9	95.2	0.66
Low income	66.6	59.3	0.69	78.2	81.3	0.33	80.6	78.6	0.79
Marriage	45.4	38.8	0.7	53.7	53.7	0.1	78.1	66.8	0.19

All data are presented as percentages unless specified otherwise. Continuous variables were tested using two sample t-test and categorical variables using chi-square test. *CVA* Cerebrovascular accident, *MI* Myocardial infarction, *BMI* Body mass index

On univariable ordinal regression analysis (Table 3), female gender was significantly associated with the presence of knee pain in both HAS and KHANES groups (odds ratio 5.17[95% CI, 1.13–23.55] and 3.16[95% CI, 1.81–5.55], respectively). While non-smokers, the presence of osteoporosis, and lower education level were significantly associated with pain in univariable analysis among KNHANES subjects, they became insignificant after multivariable adjustment and only female gender remained significant. On the other hand, younger age and osteoporosis were positively and negatively associated with knee pain in OAI subjects, respectively, while only younger age remained significant after multivariable analysis (Table 3). Quality of life assessed with SF-12 was compared between non-OA, advanced OA without pain, and advanced OA with pain subjects in HAS and OAI cohorts (Table 4). Compared to non-OA subjects, advanced OA subjects without pain had comparable scores in all measures except physical function in HAS subjects, while advanced OA subjects with pain had significantly poorer scores compared to non-OA subjects in all measures except for role emotional domain. Quality of life was also comparable between non-OA and advanced OA without pain among OAI subjects. Asymptomatic advanced OA subjects had comparable WOMAC pain, stiffness, and physical function score compared to non-OA subjects in both HAS and OAI, and total WOMAC score was not significantly different between non-OA and asymptomatic OA subjects in the OAI cohort (Table 5).

**Table 3:** Risk factors associated knee pain in advanced radiographic knee OA in three study subjects

Variables	HAS(*N* = 47)		KNHANES(*N* = 685)		OAI(*N* = 554)	
	Unadjusted OR (95% CI)	Adjusted OR (95% CI)	Unadjusted OR (95% CI)	Adjusted OR (95% CI)	Unadjusted OR (95% CI)	Adjusted OR (95% CI)
Age	0.91(0.81–1.02)	NA^c^	0.91(0.81–1.02)	**–**	**0.91(0.86–0.95)**	**0.91(0.86–0.95)**
Female	**5.17(1.13–23.55)**	NA^c^	**3.16(1.81–5.55)**	**3.16(1.81–5.55)**	0.86(0.42–1.73)	–
Diabetes	1.25(0.13–12.51)	NA^c^	1.3(0.85–2)	–	0.7(0.24–2.09)	–
CVA	NA^a^	NA^c^	2.33(0.79–6.9)	–	1.06(0.14–8.25)	–
MI	NA^a^	NA^c^	1.14(0.54–2.43)	–	0.6(0.07–4.82)	–
Hyperlipidemia	NA	NA^c^	1.57(0.96–2.56)	–	NA	–
Osteoporosis	3.85(0.43–34.06)	NA^c^	**2.3(1.29–4.09)**	**–**	**0.4(0.17–0.93)**	–
BMI	1(0.86–1.17)	NA^c^	1.01(0.97–1.06)	–	1.03(0.95–1.11)	–
Smoker	1.5(0.27–8.27)	NA^c^	**0.56(0.38–0.83)**	–	1.65(0.79–3.44)	–
Alcohol	0.76(0.2–2.98)	NA^c^	0.88(0.63–1.24)	–	1.29(0.54–3.08)	–
Low education	NA^b^	NA^c^	**3.04(1.73–5.34)**	–	1.57(0.21–11.96)	–
Low income	0.73(0.15–3.46)	NA^c^	1.22(0.82–1.82)	–	0.57(0.24–1.33)	–
Marriage	0.76(0.2–2.98)		1(0.72–1.38)	–	0.88(0.35–2.21)	–

^a^Among subjects without pain, none had this condition. ^b^Among subjects without pain, all had low education level. ^c^ In HAS groups, because there were so few subjects, adjustment analysis was not possible. For Adjust in KNHANES and OAI, we used logistic regression analysis with the backward selection method. *CVA* Cerebrovascular accident, *MI* Myocardial infarction, *BMI* Body mass index

**Table 4:** Health related QOL measured by SF-12 in HAS and OAI subjects

Variables	HAS			OAI		
	Non-OA (*n* = 103)	Advanced OA without pain (*n* = 11)	Advanced OA with pain (*n* = 36)	Non-OA (*n* = 588)	Advanced OA without pain (*n* = 33)	Advanced OA with pain (*n* = 521)
Physical functioning (PF)	66.99 ± 33.43	47.72 ± 28.4*	27.85 ± 32.52*	85.31 ± 25.26	84.38 ± 25.20	69.08 ± 30.91*
Role physical (RP)	75.36 ± 32.01	70.45 ± 25.16	35.71 ± 32.81*	84.41 ± 21.40	81.25 ± 23.97	70.47 ± 25.20*
Bodily pain (BP)	82.03 ± 25.34	75 ± 27.38	41.42 ± 30.88*	82.84 ± 21.27	85.16 ± 21.87	70.13 ± 24.37*
General health (GH)	48.2 ± 24.19	44.09 ± 30.56	25.42 ± 25.15*	80.96 ± 19.06	77.66 ± 17.27	73.39 ± 19.54*
Vitality (VT)	37.62 ± 29.67	27.27 ± 23.59	18.57 ± 22.96*	65.33 ± 21.09	70.31 ± 16.11	61.03 ± 21.69*
Social functioning (SF)	87.86 ± 24.21	81.81 ± 35.51	68.57 ± 33.39*	90.32 ± 18.69	98.44 ± 8.84*	89.50 ± 19.85
Role emotional (RE)	86.04 ± 21.53	93.18 ± 16.16	72.5 ± 35.32	91.04 ± 15.60	93.75 ± 14.89	87.86 ± 19.47*
PCS	68.14 ± 24.16	59.31 ± 21.43	32.6 ± 24.25*	80.38 ± 17.72	82.11 ± 18.20	70.76 ± 20.32*
MCS	71.6 ± 18.71	67.61 ± 20.12	55.98 ± 23.01*	80.60 ± 14.59	86.13 ± 9.72	79.00 ± 14.97*

*PCS* Physical component summary, *MCS* Mental component summary. Data are presented as the mean ± SD. *: *P* value < 0.05 versus non-OA patients by Mann Whitney test within group

**Table 5:** Health related QOL measured by WOMAC score in HAS and OAI subjects

Variables	HAS			OAI		
	Non-OA (*n* = 103)	Advanced OA without pain (*n* = 11)	Advanced OA with pain (*n* = 36)	Non-OA (*n* = 588)	Advanced OA without pain (*n* = 33)	Advanced OA with pain (*n* = 521)
WOMAC pain score	53.38 ± 80.14	98.36 ± 100.03	285.69 ± 152.61	2.38 ± 3.23	1.50 ± 2.10	5.58 ± 3.83
WOMAC stiffness score	16.95 ± 33.53	20.36 ± 29.76	117.00 ± 68.67	1.39 ± 1.64	0.94 ± 1.22	3.05 ± 1.74
WOMAC function score	25.52 ± 34.65	50.00 ± 41.23	122.31 ± 59.89	6.91 ± 10.57	5.37 ± 7.95	17.77 ± 12.79
Total WOMAC score	227.11 ± 321.47	458.27 ± 424.88*	1364.97 ± 687.58*	10.55 ± 14.75	7.71 ± 10.87	26.13 ± 17.38*

HAS group used the 100 mm visual analog version and WOMAC score range was 0–2400. OAI group used the Likert scale version and score range was 0–96. Data are presented as the mean ± SD. *: *P* value < 0.05 versus non-OA patients by Mann Whitney test within group

## Discussion

In this study, we examined the prevalence of pain among subjects with advanced radiographic knee OA and clinical characteristics associated with pain using two Korean databases and one American database. The results showed that 23.5 and 31.2% of subjects with KL grade 4 OA were asymptomatic in the Korean group and 5.9% of subjects with KL grade 4 OA were asymptomatic in the American group. Female gender was significantly associated with pain. Advanced radiographic knee OA subjects without pain did not differ in terms of quality of life compared to non-radiographic OA subjects.

Although many studies have reported that radiographic knee OA is poorly correlated with knee pain, and risk factors for radiographic OA are not strong predictors of knee pain, a study of Caucasian subjects, including patients with knees discordant for the presence of pain or pain severity, showed that the severity of radiographic knee OA was strongly associated with both the presence of frequent knee pain and pain severity [24–26]. That study used a within-person, knee-matched, case-control design so that all person-level factors related to pain were distributed evenly between both knees, eliminating their confounding effects between individual subjects. These findings were corroborated by another study in an Asian population, which showed that there was a strong dose–response relationship between the severity of radiographic knee OA and frequent knee pain [27]. Compared to knees with KL grade 0, the odds ratios of frequent knee pain were 3.0–6.8, and 5.9–54.7 among knees with KL grade 2 and KL grade 3–4, respectively.

These findings indicated that the pathological changes revealed by radiographs were indeed correlated with pain. However, the within-person, knee-matched approach may have inherent limitations due to the possibility of selecting unilateral knee OA with a traumatic etiology. The fact that pain perception is a complex process related to psychosocial factors as well as joint pathology may explain the phenomenon of knee pain in the absence of knee OA. On the other hand, the absence of pain in subjects with severely damaged joints is difficult to explain with the traditional concept of nociceptive sensory input from tissue damage being the main mechanism of pain, and consistent with the concept that pain is a subjective experience unique to each individual, with natural variability among individuals in terms of sensitivity and perception.

Compared to Korean subjects, OAI subjects with advanced OA were mostly symptomatic and only 5.9% did not have knee pain. This discrepancy may have been related to the differences in study subjects, as well as selection of participants or definition of knee pain. The questionnaires for knee pain differed in the three studies (presence of knee pain lasting at least 1 month during life, or lasting more than 30 days over the past 3 months in the two Korean studies, pain on most days at least 1 month during the past 12 months in OAI), and it is possible that OAI captured pain status more broadly. Our result shows that OAI group had higher prevalence of pain compared to Korean groups. Higher BMI among OAI group compared to Korean group may result in more pain, because previous study showed that OA subjects with high BMI had a greater likelihood of knee pain compared to subjects with a normal BMI [28]. The potential impact of high BMI on pain may be mediated through inflammatory pathways, as well as difference in load bearing [29]. Although ethnic differences in the response to acute pain have been reported [30, 31], this would not fully account for the results because previous reports in Caucasian populations showed that although there was a direct relationship between the severity of radiographic OA and knee pain, only 64% with KL grade 3 had experienced pain on some occasions [32]. A more recent report showed that 25.8% of Dutch subjects with KL grade 3–4 were also asymptomatic, consistent with our results [12].

Female gender was the only factor associated with pain in Korean advanced OA subjects. This result was consistent with a previous study showing that women reported greater knee pain than men in all KL grades although the gender difference was attenuated at KL grade 4 [33]. In that study, the sex differences in knee OA symptoms were postulated to have been due to differences in hormones, body composition, psychosocial characteristics, knee structure, and neural processing. Another study suggested that enhanced central sensitivity may be an important contributor to the greater overall sensitivity to experimental pain in women compared to men [34].

In the OAI study, younger age was associated with pain. Although a range of painful diseases show higher prevalence in the elderly, older age has been reported to be both positively and negatively associated with knee pain [8, 35]. Our finding indicated that young individuals may have higher sensitivity to pain. In regression analysis to explore the risk factor associated knee pain in advanced radiographic OA in our study, we didn’t adjust for “a history of knee injury” because it was not available for KNHANES cohort. We only included variables that could be identified in all three groups. Because knee injury is a risk factor for knee pain [36], we cannot exclude the possibility that the association between young age and knee pain in the OAI cohort might have resulted from this. However, among Korean subjects, age was not associated with pain in any direction. The reason for the discrepancy in the association of osteoporosis with knee pain in the two ethnic groups remains unclear. Epidemiological studies have frequently shown an inverse association between osteoporosis and OA, with a stronger association for large joint OA. However, there is a paucity of data regarding the relationship between OA pain and osteoporosis [37]. Although osteoporosis is considered to be a major cause of musculoskeletal pain, how osteoporosis causes pain aside from pain resulting from fractures, is poorly understood. Osteoclasts resorb bone by secreting protons into the extracellular compartment, which leads to an acidic microenvironment, a well-known cause of pain [38, 39]. The relationship between osteoporosis and OA pain, and its pathogenetic mechanism is an important subject of further research.

In our study, health-related quality of life was not significantly different between non-OA and asymptomatic advanced radiographic knee OA subjects. Independent of radiographic changes, knee pain has been reported to be an important determinant of physical disability in knee OA [5, 40]. Creamer et al. reported that decreased physical activity due to pain is associated with impaired physical performance in subjects with knee OA. Therefore, it is plausible that knee pain may lead to a decreased quality of life through impairment of physical performance, independently of the presence of OA [5]. Although therapeutic trials with nerve growth factor suggests that pain control may lead to accelerated degradation of joints, it is also possible that proper control of pain may help to retard the physical disability in OA.

Our study had some limitations. Due to the heterogeneity of data collection methods used, the data could not be combined and analyzed. Some of the discrepancies between cohorts may have been due to the differences in data collection methodologies, such as the questionnaire for knee pain. The definition of asymptomatic knee OA subjects as those who responded “no” to the screening knee pain questionnaire is arbitrary, and may have missed subjects with milder degrees of knee pain. Finally, we did not include patello–femoral OA because KL grade 4 only relates to tibiofemoral OA. How advanced patellofemoral OA contributes to knee pain would be an important research subject.

## Conclusions

The results of the present study indicated that 5.9–31% of subjects with radiographic KL grade 4 OA derived from 3 different study populations did not have knee pain Therapeutic decision-making based simply on imaging studies, as well as treatment options focusing solely on cartilage engineering, should be viewed with caution.

## Supplementary information

**Additional file 1.** Questionnaires survey in three groups.

## Data Availability

The datasets used and analyzed during the current study are available from the corresponding author on reasonable request.

## Abbreviations

AP: Anteroposterior
BMI: Body mass index
BP: Bodily pain
CI: Confidence intervals
CVA: Cerebrovascular accident
GH: General health
HAS: Hallym aging study
IQR: Interquartile range
KL: Kellgren–Lawrence
KNHANES: Korean national health and nutrition examination survey
MCS: Mental component summary
MI: Myocardial infarction
OA: Osteoarthritis
OAI: Osteoarthritis initiative
OR: Odds ratio
PA: Posterior–anterior
PCS: Physical component summary
PF: Physical functioning
RE: Role emotional
RP: Role physical
SD: Standard deviations
SF: Social functioning
THR: Total hip replacement
TIA: Transient ischemic attack
TKR: Total knee replacement
VT: Vitality
WOMAC: Western Ontario & McMaster Universities
YLD: Years lived with disability
