# Supplementary material for: Absence of pain in subjects with advanced radiographic knee osteoarthritis
Source: BMC Musculoskelet Disord. 2020 Sep 29;21:640. doi: 10.1186/s12891-020-03647-x (PMC7526196; doi:10.1186/s12891-020-03647-x)
Supplement: Supplementary file 1 — Additional file 1. Questionnaires survey in three groups. [file 12891_2020_3647_MOESM1_ESM.pdf]

Supplementary table 1. Questionnaires survey in three groups.

| Questionnaires survey | HAS | KNHANES | OAI |
|---|---|---|---|
| CVA | Q. Have you ever been diagnosed with a stroke? | Q. Have you ever been diagnosed with a stroke by a doctor? | Q. had stroke, cerebrovascular accident, blood clot or bleeding in brain, or transient ischemic attack (TIA)? |
| Myocardial infarction | Q. Have you ever been diagnosed with an angina or myocardial infarction? | Q. Have you ever been diagnosed with an angina or myocardial infarction by a doctor? | Q. ever had heart attack |
| Diabetes mellitus | Q. Have you ever been diagnosed with diabetes mellitus? | Q. Have you ever been diagnosed with diabetes mellitus by a doctor? | Q. had diabetes (high blood sugar) |
| Hyperlipidemia | Q. Have you ever been diagnosed with hyperlipidemia? | Q. Have you ever been diagnosed with hyperlipidemia by a doctor? | NA |

| | | | |
|---|---|---|---|
| Osteoporosis | Q. Have you ever been diagnosed with osteoporosis? | Q. Have you ever been diagnosed with osteoporosis by a doctor? | Q1. taken bisphosphonate medication(includes alendronate, risedronate) to treat osteoporosis or paget disease past 5 years<br><br>Q2. Used parathyroid hormone or PTH (includes Forteo, or teriparatide, given by injection) to treat osteoporosis past 6 months. |

HAS: Hallym Aging Study, KNHANES: the Korean National Health and Nutrition Examination Survey, OAI: Osteoarthritis Initiative, CVA: cerebrovascular accident
